# In this month’s Bulletin

**DOI:** 10.2471/BLT.21.000721

**Published:** 2021-07-01

Aquiles R Henriquez-Trujillo et al. (478) describe the impact of COVID-19 outbreaks among Amazonian indigenous people, in Ecuador. Mohammad I Khan et al. (479) make the case for increased vaccine manufacturing capacity in low- and middle-income countries.

Gary Humphreys and Lynn Eaton (482–483) report on adaptions made to clinical trials to ensure faster vaccine development for COVID-19. Renu Khanna talks to Andréia Azevedo Soares (484–485) about improving policies for women’s health.

## Burkina Faso, Cambodia, Canada, Democratic Republic of the Congo, Denmark, Ethiopia, Ghana, Guinea-Bissau, India, Iran (Islamic Republic of), Iraq, Myanmar, Nepal, New Zealand, Niger, Nigeria, Norway, Senegal, Sierra Leone, Uganda, United Kingdom of Great Britain and Northern Ireland, United Republic of Tanzania, United States of America, Zambia

### Trauma, burns, cardiac arrest, opioid poisoning, malaria, paediatric communicable diseases and malnutrition

Aaron M Orkin et al. (514–528) review the evidence for provision of emergency care by lay responders.

## Japan

### SARS-CoV-2 dynamics during shipboard quarantine

Ting-Yu Yeh & Gregory P Contreras (486–495) track viral evolution.

## Pakistan

### Preventing rabies

Naseem Salahuddin et al. (506–513) study a shorter post-exposure prophylaxis regimen.

## Russian Federation

### Screening for alcohol use 

Maria Neufeld et al. (496–505) adapt and validate WHO’s questionnaire.

## Global

### Complex socioeconomic models needed 

Pier Luigi Sacco & Manlio De Domenico (529–535) compare COVID-19 to previous shocks.

### National focal points for the *International Health Regulations*

Kumanan Wilson et al. (536–538) argue for better role recognition and support.

### Marketing tobacco in African countries

Eric Crosbie et al. (539–540) review supply and demand strategies.

